# System Analysis of ROS-Related Genes in the Prognosis, Immune Infiltration, and Drug Sensitivity in Hepatocellular Carcinoma

**DOI:** 10.1155/2021/6485871

**Published:** 2021-11-08

**Authors:** Jun Hui Xu, Yong Jun Guan, Zhen Dong Qiu, Xin Zhang, Liu Liu Zi, Yu Zhou, Chen Chen, Jia Yu, Yi Chao Zhang, Wei Xing Wang

**Affiliations:** ^1^Department of Hepatobiliary Surgery, Renmin Hospital of Wuhan University, Wuhan, China; ^2^Central Laboratory, Renmin Hospital of Wuhan University, Wuhan, China; ^3^Hubei Key Laboratory of Digestive System Disease, Wuhan, China

## Abstract

Hepatocellular carcinoma (HCC) is an aggressive malignant tumor with a poor prognosis. Reactive oxygen species (ROS) play an important role in tumors; however, the role of ROS-related genes is still unclear in HCC. Therefore, we analyzed the role of ROS-related genes in HCC via bioinformatics methods. Firstly, a prognosis model was constructed using LASSO Cox regression and multivariate analyses. We also investigated the potential function of the ROS-related genes and the correlation with immune infiltration, tumor stemness, and drug sensitivity. ICGC database was used for validation. Secondly, we further analyzed the role of 11 ROS-related genes in HCC. As a member of ROS gene family, the role of STK25 has remained unclear in HCC. We explored the biological function of STK25 using *in vitro* experiments. The present study was the first to construct a ROS-related prognostic model in HCC. The correlation of ROS-related genes with immune infiltration, tumor stemness, and drug sensitivity was dissected. Furthermore, we demonstrated that STK25 knockdown could increase the proliferation, migration, and invasion capacity of HCC cells.

## 1. Introduction

Hepatocellular carcinoma (HCC) is the primary pathological type of liver cancer and is one of the most common malignancies worldwide [1, 2]. Liver cancer ranks the second leading cause of cancer-related death due to lack of effective treatment [3]. Currently, the mainstay treatment of HCC is surgical excision, liver transplantation, interventional, chemoradiotherapy, and targeted drug therapy. The early diagnosis of HCC is difficult, and hence, the majority of HCC patients suffer from a poor prognosis with a high recurrence rate. Therefore, it remains clinically essential to identify the novel and effective diagnostic markers for HCC. Chronic liver diseases, such as hepatitis B virus, liver cirrhosis, alcoholic liver disease, and nonalcoholic fatty liver disease, are the major risk factor for HCC [4]. HCC is a highly heterogeneous disease, and its mechanism of HCC is still not completely understood. Abnormal expression and mutation of genes contribute to the progression of HCC [5]. However, the underlying mechanisms of HCC development and the key driving factors of carcinogenesis are still unclear, which impedes the development of targeted treatment [6].

Reactive oxygen species (ROS) are regarded as reactive oxygen metabolites and oxygen-containing materials, including superoxide anion (O_2_-) and hydroxyl radical (OH_-_) as well as nonradical molecules, such as hydrogen peroxide (H_2_O_2_) [7]. At normal concentrations, ROS serves as the second message that participates in a diversity of signal transduction and regulates cell growth, differentiation, and proliferation. Nevertheless, oxidative stress is the consequence of the imbalanced redox state accompanied by ROS production exceeds cell capacity for ROS scavenging that has been implicated in HCC occurrence [8]. Previous studies have indicated that ROS play a vital role in the progression of HCC through the induction of autophagy [7]. However, a comprehensive analysis of the role of ROS-related genes in HCC has not been reported.

Serine/threonine-protein kinase 25 (SK25), also known as YSK1 or SOK1, plays important roles in different biological processes, such as the regulation of cell migration and modulation of Golgi morphology [9–12]. Some studies have shown that SOK1 can be activated by chemical anoxia induction, which is dependent upon the generation of ROS [13, 14]. In addition, a previous research has indicated that SOK1 promotes the apoptotic response to ROS with marked ROS production and severe ATP depletion [12]. However, as an ROS-related gene, the role of STK25 in liver cancer has not been reported.

In the present study, we systematically investigated the expression and clinicopathological characteristics of ROS-related genes and constructed ROS-related gene prognostic model in HCC patients. We further demonstrated the relationship between ROS-related genes and tumor-infiltrating immune cells. An ROS-related gene risk model can be used as a prognostic biomarker to predict immune microenvironment in HCC patients. Moreover, focusing on the clinicopathological and immunological characteristics of ROS-related genes in HCC may optimise tumor immunotherapy.

## 2. Materials and Methods

### 2.1. Public mRNA Expression Datasets

The mRNA expression of 371 HCC patients and the clinicopathological information of HCC samples were extracted from TCGA (https://portal.gdc.cancer.gov/) and ICGC (https://dcc.icgc.org) databases. In TCGA database, among the 371 patients, six patients were excluded due to lack of survival time, and 365 patients were eventually included in the study (Table 1). All the raw count data were analyzed to identify differential expression genes (DEGs) in HCC samples and matched noncancerous samples by the package “limma” of the R software. ∣log2FC | = 0 and *P* < 0.05 were set as the cut-off point. The mRNA data and clinical information of 231 liver cancer samples were downloaded from the ICGC database (http://dcc.icgc.org/projects/LIRI-JP). Then, we summarized 49 ROS-related genes from the Molecular Signatures Database (MSigDB) v7.2 (https://www.gsea-msigdb.org/gsea/msigdb/index.jsp) [15] for further analysis. They were listed in Supplementary Table S1. 38 detailed immune checkpoint genes are listed in the Supplementary Table S2.

### 2.2. Construction of a ROS-Related Gene-Based Signature

All the raw data were analyzed by the “limma” package in the R software and identified the DEGs between HCC tissues and noncancerous tissues. Univariate regression analysis of OS was conducted to screen ROS-related prognostic genes. Benjamini & Hochberg (BH) correction was used to adjust the *P* value. Then, the STRING database (version11.0) was used for constructing overlapping differential prognostic gene interaction networks [16]. In order to minimize the risk of overfitting, LASSO-penalized Cox regression analysis was used to develop the prognosis model [17, 18]. The LASSO algorithm was used for variable selection, combined with “glmnet” R package for shrinkage. In the regression model, we took the normalized expression matrix of the candidate differential prognostic genes as the independent variable and the overall survival and state of the patients in TCGA cohort as the response variable. The penalty parameter (model parameter) of the model is calculated by ten ties cross-validation according to the minimum standard. The risk score was calculated based on the normalized expression level of each gene and its corresponding regression coefficient. The coefficient of each gene is listed in Supplementary Table S3. The formula was built as follows: score = *e*^sum^ (each gene′s expression × corresponding coefficient). Patients were classified into high-risk and low-risk groups depending on the median risk score. Principal component (PCA) analysis was performed using “PRCOMP” function of “stats” R package based on the expression of gene signature. In addition, the “Rtsen” R package was adopted for t-distributed stochastic neighbour embedding (t-SNE) analysis to investigate the distribution of diverse groups. Kaplan-Meier curves were utilized to estimate the differences in OS between the two groups. The time-dependent ROC curve was plotted to illustrate the sensitivity and predictive ability of gene signature based on the “survival valroc” R package.

### 2.3. The Construction of Nomograms

Nomogram models were constructed based on the expression of ROS-related prognostic genes by using the “rms” and “survival” packages in R [19]. Then, calibration curves were plotted to estimate the consistency between actual and predicted survival.

### 2.4. Functional Enrichment Analysis

To explore the potential functional features of ROS-related genes in HCC, we performed Kyoto Encyclopedia of Genes and Genomes (KEGG) and Gene Ontology (GO) analyses based on ROS-related DEGs (∣log2FC | ≥1, FDR < 0.05) between different risk groups via the “clusterProfiler” R package.

### 2.5. Immune Infiltration Analysis

Then, we adopted the “gsva” R package to investigate single-sample gene set enrichment analysis (ssGSEA) and to calculate the relationship between risk score and 16 immune-infiltrating cells and 13 immune-related pathways. We further explored the connection between ROS-related DEGs and tumor purity and immune-infiltrating cells by the TIMER and TISIDB databases based on a previously published statistical deconvolution method from gene expression profiles. Tumor Immune Estimation Resource (TIMER) (http://cistrome.org/TIMER/) [20] is an ideal resource that contains 10897 samples across 32 cancer types from TCGA, and it can be adopted to comprehensively analyze immune infiltration levels of diverse cancer types. TISIDB (http://cis.hku.hk/TISIDB/) is also an online web with different types of data which can be used to explore the relationship between the tumor and immune infiltration [21].

### 2.6. The GEPIA

GEPIA (Gene Expression Profiling Interactive Analysis) (http://gepia.cancer-pku.cn/) has been a valuable and highly cited resource for gene expression analysis based on tumor and normal samples from TCGA and the GTEx databases [22]. GEPIA2021 is a standalone extension with multiple deconvolution-based analyses for GEPIA. They deconvolute each sample tool by TCGA/GTEx with the bioinformatics tools CIBERSORT, EPIC, and quanTIseq [19].

### 2.7. The UALCAN Analysis

UALCAN is a comprehensive, user-friendly, and interactive web resource for analyzing cancer OMICS data. It is built on PERL-CGI with high-quality graphics using JavaScript and CSS [23]. UALCAN can be used to explore the expression profile and patient survival information for genes and to evaluate epigenetic regulation of gene expression by promoter methylation.

### 2.8. The Kaplan-Meier Plotter Analysis

The Kaplan-Meier plotter can be used to estimate the effect of 54 k genes on survival in 21 cancer types including liver cancer (*n* = 365) [19, 24]. Sources for databases are from GEO, TCGA, and EGA.

### 2.9. Clinical Sample Collection

Nine HCC tissues and paired noncancerous tissues were collected from HCC patients who underwent liver resection in Renmin Hospital of Wuhan University (Wuhan, China) from October 2019 through October 2020. The collected tissues were stored at -80°C for subsequent use. All patients were given informed consent before surgery treatment, and the study was approved by the Ethics Committee of Wuhan University.

### 2.10. Cell Culture and Transfection

HepG2 and SMMC-7721 cells were purchased from ATCC. HepG2 cells were cultured in DMEM high glucose medium (Servicebio), and SMMC-7721 cells were cultured in 1640 medium (Servicebio), all containing 10% Foetal Bovine Serum (FBS) and 1% penicillin and streptomycin (Servicebio). Short hairpin (sh)RNAs sh-STK25 were obtained from miaolingbio.lnc, Wuhan, China. HepG2 and SMMC-7721 cells were cultured in 6-well plates (5 × 10^5^/well) and transfected with 2.4 *μ*g siRNA using Attractence Transfection Reagent (Cat. No. 301005, QIAGEN, China).

### 2.11. Immunohistochemical Staining (IHC)

This part of the operation refers to a previous study [25]. Paraffin-embedded liver cancer and corresponding noncancerous tissues were sliced and dewaxed, then treated with 3% H_2_O_2_ for 10 min to inactivate endogenous peroxidase, and treated with 0.01% mol/L sodium citrate buffer in boiling water for antigen repair. Goat serum was dropped for a block for 20 min, and STK25 antibody (1 : 50) was dropped for overnight incubation at 4°C and washed with PBS three times. Then, DAB chromogen was added. Hematoxylin was redyed, and neutral gum was sealed. The primary antibody is as follows: anti-STK25 mouse monoclonal antibody (1 : 100; Cat. No. sc-271196; Santa Cruz Biotechnology, Inc.). The sections were observed under light microscopy, and five randomized microscopic views of 200-fold magnification of each section were observed and scored.

### 2.12. Cell Proliferation Assay

The treated cells (HepG2 and SMMC-7721) were collected, and 2000 cells/well were inoculated in 96-well plates. After incubation for 24, 48, and 72 hours, 10 *μ*l CCK8 solutions were added to each well. Then, the cells were incubated at 37°C for 1 h in the dark room, and the absorbance value (OD450) of each well at 450 nm was detected by a microplate analyzer.

### 2.13. Cell Migration Assay

HepG2 and SMMC-7721 cells were seeded in 6-well plates and transfected with control plasmid and shRNA-STK25 plasmid, respectively. When the cells have grown and fused to about 90%, a sterile 200 *μ*l pipette tip was used to create an artificial wound. Then, the cells were washed three times with 1X PBS buffer to remove floating cells and replaced with a serum-free medium. Photos were taken at 0 and 24 hours under a light microscope (magnification, ×40). The results of cell migration were analyzed by the Image J software.

### 2.14. Cell Invasion Assay

The transfected HepG2 and SMMC-7721 cells were collected, resuspended in 100 *μ*l serum-free medium, and 5 × 10^4^ cells were seeded into 24-well upper transwell chamber (8 *μ*m pore size) with Matrigel (BD, USA). The lower part of the chamber was full of a medium containing 10% FBS. Invasion culture periods were about 24 h.

Cells on the top surface of the filters were wiped off using a cotton swab. The cells on the lower surface of the filter were fixed with 4% paraformaldehyde and stained with 0.1% crystal violet at 37°C for 30 min. The numbers of invading cells were counted using a light microscope with ×200 magnification.

### 2.15. Flow Cytometry Analysis

After transfection for 72 h, HCC cells were collected and washed three times with cold PBS solution. According to the instructions, HCC cells were resuspended with 500 *μ*l Annexin V binding buffer with adding 5 *μ*l Annexin V-FITC and 5 *μ*l PI, following incubation at room temperature for 30 min, and flow cytometer was performed to analyze apoptosis.

### 2.16. EdU Assay

HepG2 and SMMC-7721 cells with appropriate concentrations were added to the 6-well plates with 2.4 *μ*g plasmids (empty vector and shRNA-STK25). EdU working solution (10 *μ*M) was added into cells in 6-well plates and incubated for 2 hours. After that, the culture medium was removed, and 1 ml 4% paraformaldehyde was added to each well at room temperature for 15 min. Then, the cells were washed with a washing solution (PBS solution containing 3% bovine serum albumin (BSA)) three times. The permeability solution (0.3% Triton X-100 solution) was added and incubated for 15 min at room temperature. Then, 0.5 ml Click reaction solution was added to each well and incubated for 30 min in the dark room. Finally, 1 ml 1X Hoechst33342 solution was added to 6-well plates and incubated for 10 min in the dark room. The staining was observed under a microscope.

## 3. Statistical Analysis

Student's *t*-test was employed to compare gene expression between HCC tissues and noncancerous tissues. Chi-square test was used to compare proportions between the high-risk group and the low-risk group. The Mann-Whitney test was applied to compare the ssGSEA score of immune cells or pathways among different risk groups. The OS in different groups was performed by Kaplan-Meier (KM) survival analysis with a log-rank test. Univariate and multivariate analyses were used to evaluate independent risk factors for OS. The association of immune checkpoint genes with the risk score was performed by R. *F*-test (one-way ANOVA) was utilized to identify the expression of 11 ROS-related genes in immune cells between tumor tissues and normal tissues. All statistical analyses were conducted using R software 3.4.2 and SPSS 23.0 software, and *P* < 0.05 was considered statistically significant.

## 4. Results

**Figure 1:**
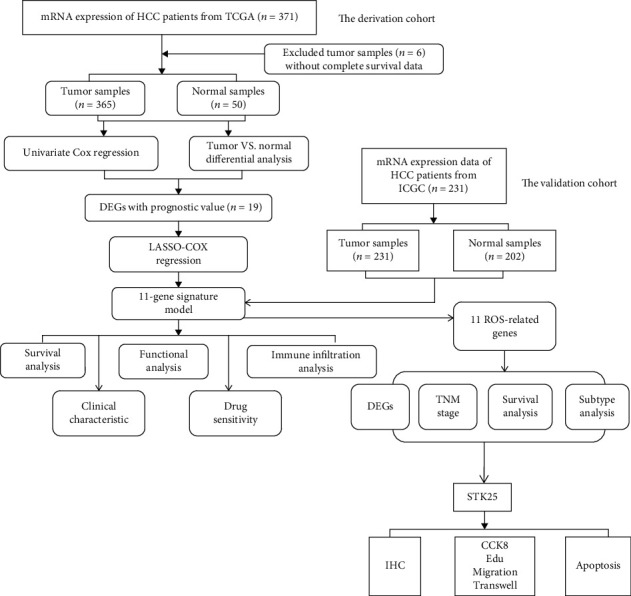
The workflow of the present study.

The workflow of the present study is shown in Figure 1. mRNA and clinical data of 371 patients with HCC were downloaded from TCGA. A total of 365 HCC patients had complete survival data. 49 ROS-related genes (Supplementary Table S1) were extracted from the MSigDB database v7.2. DEG screening analysis found that 38 ROS-related genes were differentially expressed between HCC tissues and paired noncancerous tissues. We identified 19 differentially prognostic genes by performing univariate analysis for OS. Using LASSO Cox regression and multivariate analysis, 11 ROS-related genes were identified. Data downloaded from the ICGC database were used to verify the accuracy of the model. We further conducted functional enrichment and immune infiltrating analyses. Differential expression analysis and survival analyses were performed on 11 ROS-related genes, and the relationship between the expression of the genes and TNM stages was calculated. In addition, we explored the expression of 11 ROS-related genes among different molecular and immune subtypes. Finally, only G6PD and STK25 were retained with *P* < 0.05. Although, the role of G6PD has been reported in HCC, the role of STK25 has remained unclear. *In vitro* experiments were carried out to explore the role of STK25 in liver cancer.

### 4.1. Identification of Prognostic ROS-Related DEGs in TCGA Datasets

**Figure 2:**
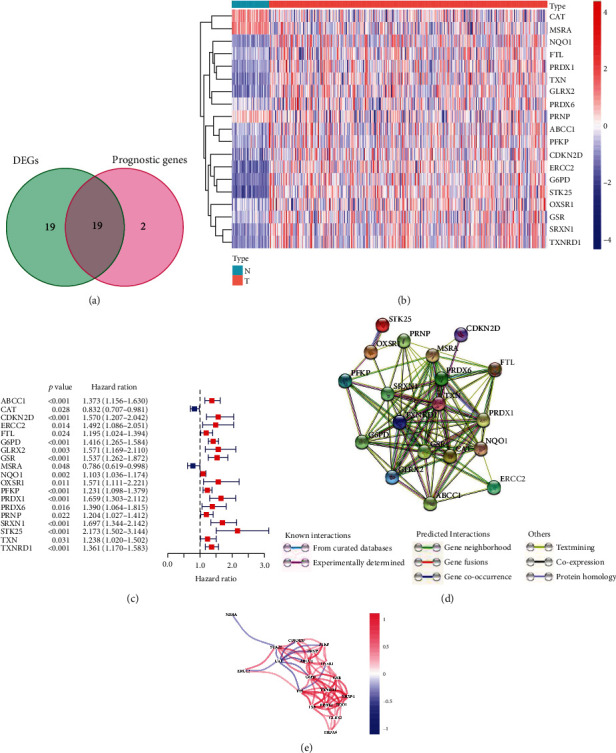
Identification of the candidate ROS-related genes in TCGA database. (a) Identification of differentially expressed genes in HCC tissues and paired noncancerous tissues that were related to OS. (b) 19 Overlapping genes were overexpressed in HCC tissues. (c) Forest plots indicating the results of the univariate Cox regression analysis between OS and gene expression. (d) The PPI network downloaded from the STRING database revealed the interactions among the DEGs. (e) The correlation network of DEGs. The correlation coefficients are denoted by different colors.

49 ROS-related genes listed in Supplementary Table S1 were drawn from the MSigDB database. 38 ROS-related genes were differentially expressed between HCC tissues and paired noncancerous tissues by DEG screening analysis based on the “limma” package in the R software. In addition, based on TCGA database, we identified 19 differentially prognostic genes by performing univariate analysis for OS. The results of univariate regression analysis are shown in Figures 2(a)–2(c) (*P* < 0.05). The genes included are CAT, MSRA, NQO1, FTL, PRDX1, TXN, GLRX2, PRDX6, PRNP, ABCC1, PFKP, CDKN2D, ERCC2, G6PD, STK25, OXSR1, GSR, SRXN1, and TXNRD1. Based on the result of univariate analysis, a protein-protein interaction (PPI) network was built to analyze the association between 19 ROS-related genes, and the finding revealed that STK25 is one of the hub genes (Figures 2(d) and 2(e)).

### 4.2. Constructing a ROS-Related DEG Prediction Model in TCGA Datasets

**Figure 3:**
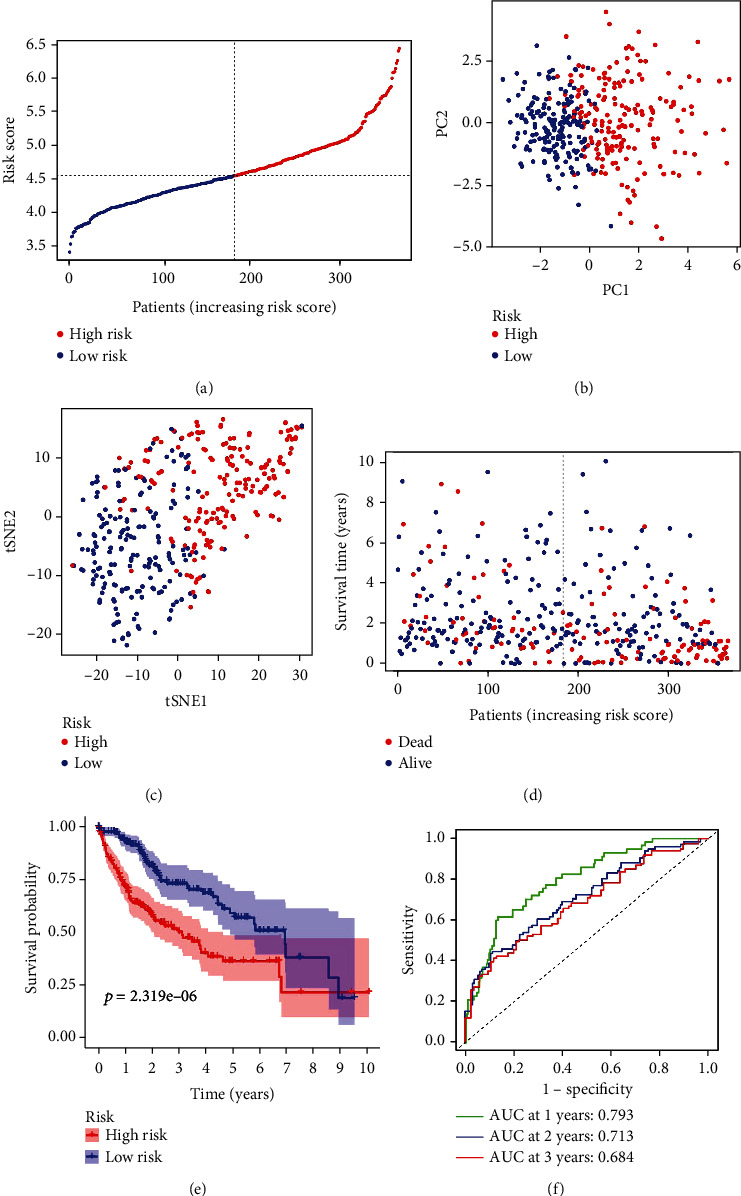
Prognostic analysis of 11-gene signature models in TCGA database. (a) The distribution and median value of the risk score in TCGA database. (b) PCA plot of TCGA database. (c) t-SNE analysis of TCGA database. (d) The distribution of OS status, OS, and risk score in TCGA database. (e) The OS of HCC patients between the high-risk group and low-risk group were analyzed by Kaplan-Meier curves. (f) AUC of time-dependent ROC curves confirmed the prognostic value of the risk score in TCGA database.

**Figure 4:**
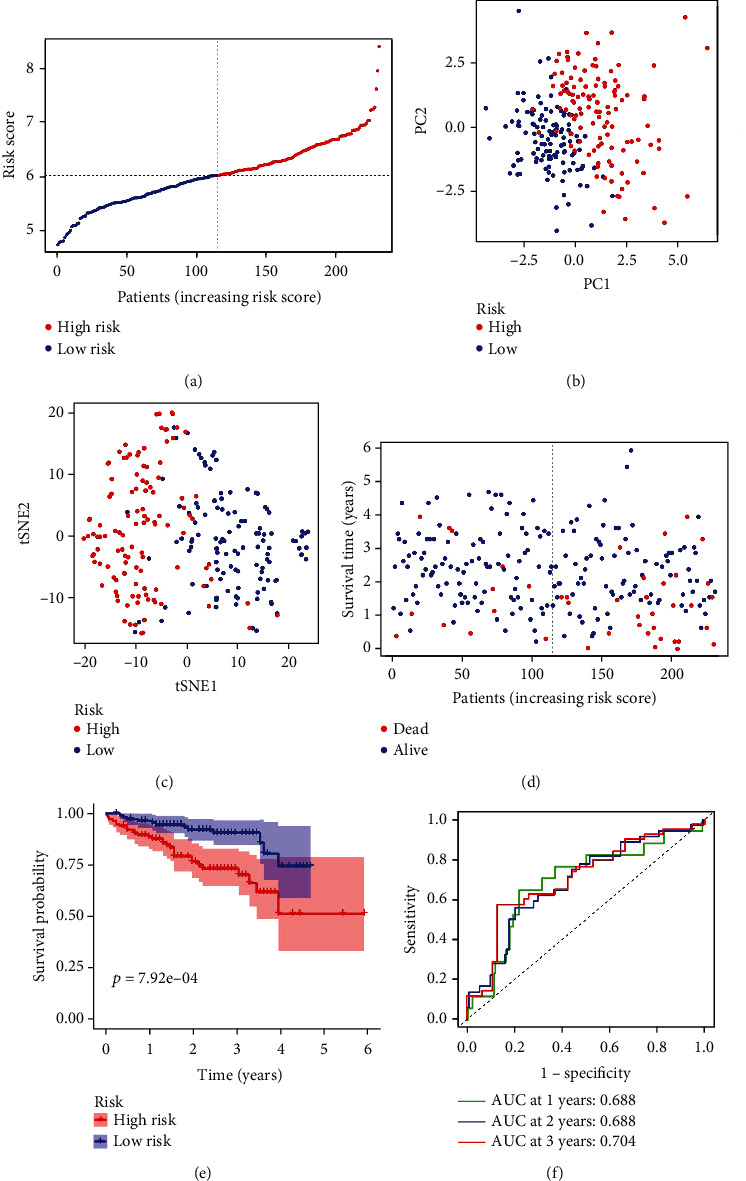
Prognostic analysis of 11-gene signature models in the ICGC database. (a) The distribution and median value of the risk score in the ICGC database. (b) PCA plot of the ICGC database. (c) t-SNE analysis of the ICGC database. (d) The distribution of OS status, OS, and risk score in the ICGC database. (e) The OS of HCC patients between the high-risk group and low-risk group was analyzed by Kaplan-Meier curves. (f) AUC of time-dependent ROC curves confirmed the prognostic value of the risk score in the ICGC database.

LASSO Cox regression was performed to construct a prognostic model, and based on TCGA database, 11 ROS-related genes were retained by the coefficient values, which include CDKN2D, G6PD, GLRX2, GSR, MSRA, OXSR1, PFKP, PRDX1, PRDX6, SRXN1, and STK25. Patients were dichotomized into high and low groups based on the median cut-off value. Patients in the high-risk group were linked to tumor grade (*P* < 0.001) (Table 2). Next, we performed PCA and t-SNE to explore the distribution of patients with different risk scores. The results showed that patients were grouped into two directions, and the high-risk group had a shorter survival time than the low-risk group (*P* < 0.05) (Figures 3(a)–3(d)). This was supported by the results of Kaplan-Meier analysis, which showed that the OS time was shorter in the high-risk group compared with the low-risk group (*P* = 2.319*e* − 06) (Figure 3(e)). Furthermore, ROC further demonstrated the possibility and accuracy of OS prediction based on the risk score. The AUC values of 1-year survival, 3-year survival, and 5-year were 0.793, 0.713, and 0.684, respectively (Figure 3(f)). Next, based on the ICGC database, we performed LASSO Cox regression, PCA, t-SNE, survival analysis, and ROC curve to demonstrate the aforementioned results. Our findings indicated that the high-risk group had a shorter survival time than the low-risk group, and the low-risk group had a longer survival time than the high-risk group (*P* < 0.05) (Figures 4(a)–4(d)). Survival analysis revealed the high-risk group had a shorter OS time than the low-risk group (*P* < 0.05) (Figure 4(e)). The results of ROC manifested that the AUC values of 1-year survival, 2-year survival, and 3-year survival were 0.793, 0.713, and 0.704, respectively (Figure 4(f)). In the ICGC database, only a few liver cancer patients showed 5-year survival, and no results were obtained with R analysis. The ROC of 1, 2, and 3 years was performed to confirm the diagnostic value of the model.

### 4.3. Prognostic Significance of the 11-Gene Signature in HCC

**Figure 5:**
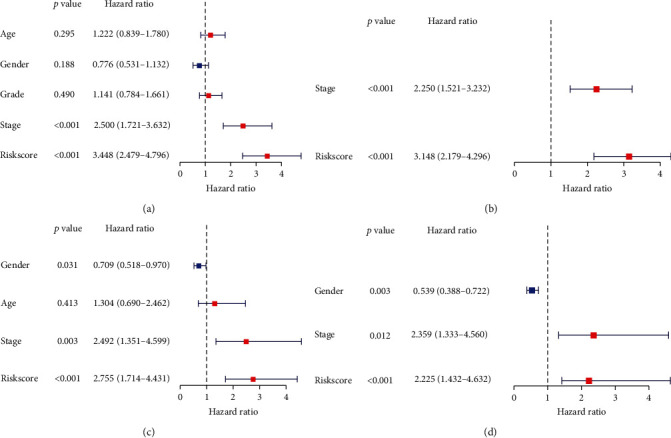
Results of the univariate and multivariate Cox regression analyses regression regarding OS. (a) The result of univariate Cox regression in TCGA database. (b) The result of multivariate Cox regression in TCGA database. (c) The result of univariate Cox regression in the ICGC database. (d) The result of multivariate Cox regression in the ICGC database.

Patients were grouped into two groups (high-risk and low-risk) based on the median risk value. According to the univariate and multivariate Cox regression analyses of TCGA database, the 11-gene signature was an independent prognostic factor for OS. The risk scores of univariate regression and multivariate analyses of OS were HR = 3.448 (2.479-4.796) (*P* < 0.001) and HR = 3.145 (2.179-4.296) (*P* < 0.001) (Figures 5(a) and 5(b)). Then, we performed the univariate and multivariate Cox regression analyses based on the ICGC cohort. The results also verified that the 11-gene signature was an independent prognostic factor for OS. The risk scores of univariate and multivariate analyses of OS were HR = 2.755 (1.714-4.431) (*P* < 0.001) and HR = 2.225 (1.432-4.632) (*P* < 0.001) (Figures 5(c) and 5(d)).

### 4.4. Validation of the Prognostic Value of ROS-Related DEGs in HCC Based on Nomograms

**Figure 6:**
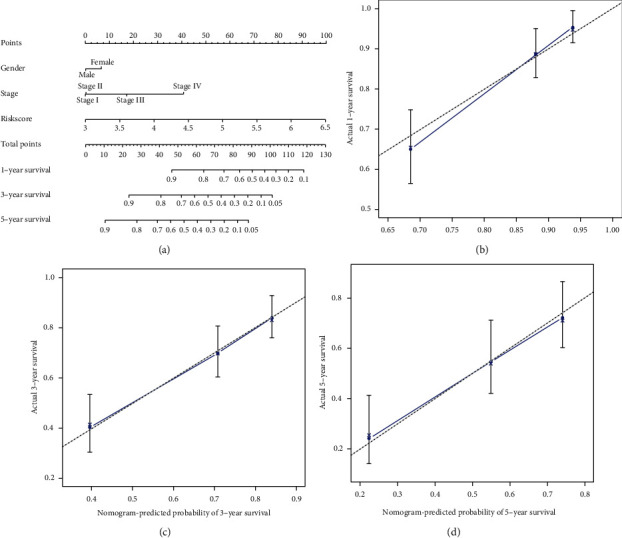
The prognostic value of nomograms for patients with HCC was based on TCGC database. (a) The calibration curve of nomograms for predicting overall survival (OS) at (b) 1 year, (c) 3 years, and (d) 5 years. The *x*-axis represents the possible OS, and the *y*-axis represents the actual OS.

To further confirm the prognostic value of ROS-related DEGs in HCC, we constructed nomograms to predict 1-year OS, 3-year OS, and 5-year OS based on the above DEGs and calculated 1-, 3-, and 5-year OS of HCC patients (Figure 6(a)). The calibration curves fitted the data well. The C-index of a nomogram for OS prediction was 0.697 (95% CI: 0.674-0.721) (Figures 6(b)–6(d)).

### 4.5. Functional Analysis in HCC

**Figure 7:**
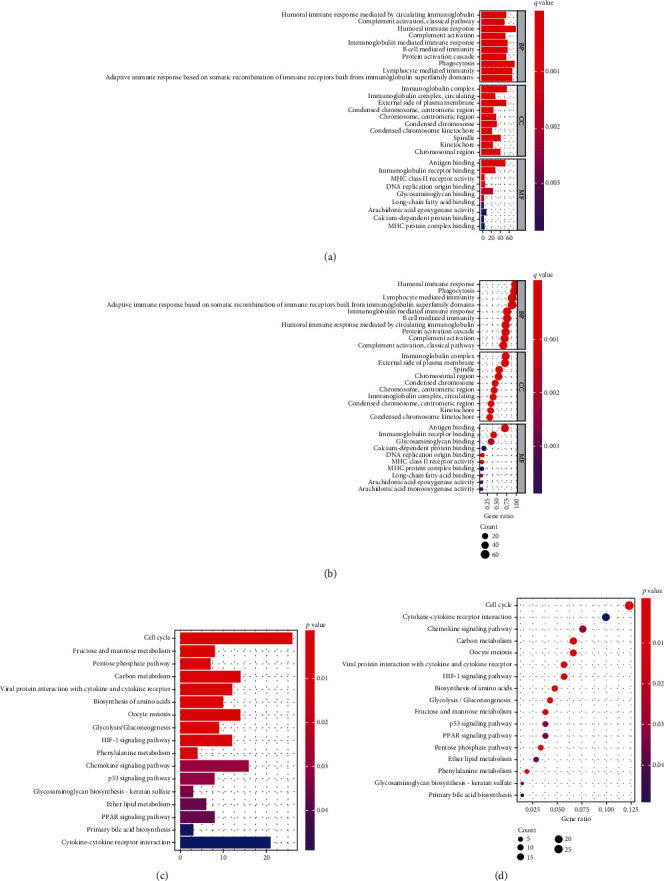
GO and KEGG analyses. (a, b) The results of GO enrichment analyses. (c, d) The result of KEGG enrichment analyses.

**Figure 8:**
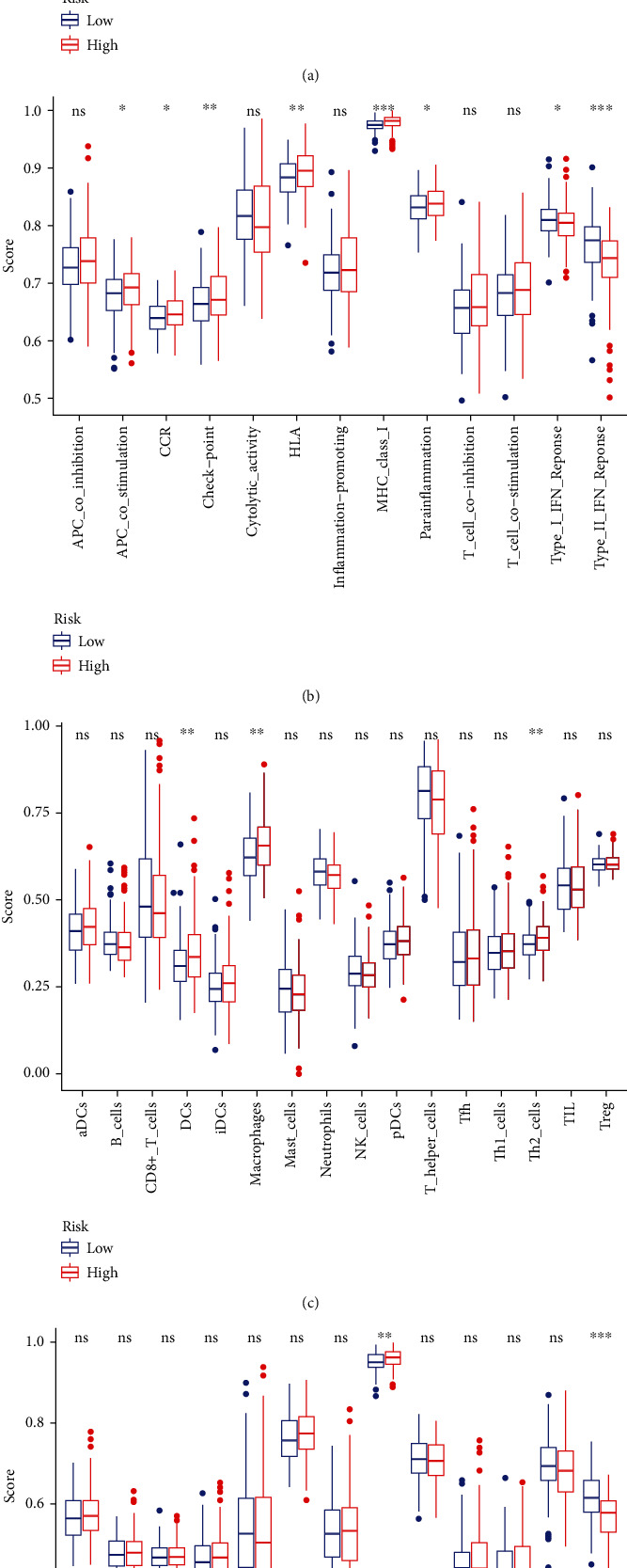
Comparison of ssGSEA score between different risk groups in the public database. (a) The score of 16 immune cells in TCGA database. (b) 13 immune-related functions are showed in boxplots. CCR: cytokine-cytokine receptor in TCGA database. (c) The score of 16 immune cells in the ICGC database. (d) 13 immune-related functions are showed in boxplots. CCR: cytokine-cytokine receptor in the ICGC database. Adjusted *P* values were displayed as follows: ns: not significant; ^∗^*P* < 0.05; ^∗∗^*P* < 0.01; ^∗∗∗^*P* < 0.001.

In addition, we determined the potential mechanisms of 11 ROS-related genes in HCC based on TCGA and ICGC databases. The “clusterProfiler” R package was adopted to conduct GO and KEGG enrichment analyses. GO analysis indicated that B cell-medicated immunity, lymphocyte-mediated immunity, immunoglobulin-mediated immune response, histocompatibility complex (MHC) class II receptor activity, and major MHC protein complex binding were enriched. KEGG analysis showed that the chemokine signaling pathway and cytokine-cytokine receptor interaction were enriched (Figures 7(a)–7(d)). Next, we also explored the relationship between risk score and immune status, and ssGSEA was used to quantify enrichment score for different subsets of immune cells as well as related functions and pathways. Our results also showed that the scores of aDCs, iDCs, macrophages, Tfh, Th1_cells, Th2_cells, and Treg in the high-risk group were higher compared with the low-risk group (Figure 8(a)). Immune infiltration of HCC with 11 ROS-related DEGs and the scores of APC_co_stimulation, CCR, checkpoint, HLA, and parainflammation were higher in the high-risk group than that in the low-risk group. The scores of type_I_IFN_response and type_II_IFN_response were lower in the high-risk group than that in the low-risk group (Figure 8(b)). Consistent with the aforementioned results, the association between risk score and immune status was investigated using the ICGC database. Our findings indicated that the scores of DCs, macrophages, and Th2_cells were higher in the high-risk group than in the low-risk group (Figure 8(c)). The score of MHC_class_I was higher, and that of type_II_IFN_response was lower in the high-risk group than in the low-risk group (Figure 8(d)).

### 4.6. Correlation of Risk Score with the Expression of Immune Checkpoint Genes for Liver Cancer

**Figure 9:**
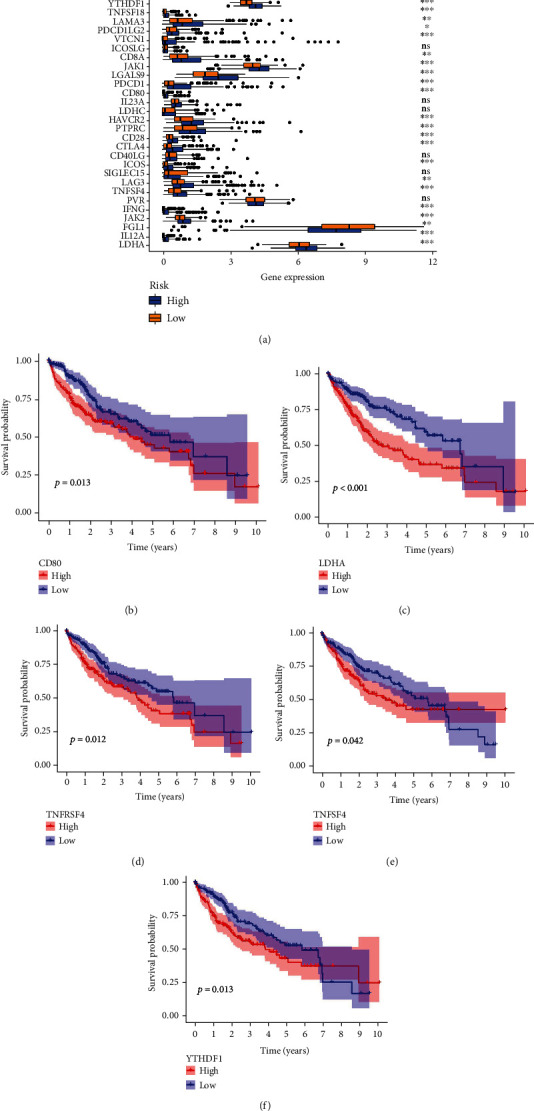
The analysis of immune checkpoint genes in HCC. (a) The expression of 38 immune checkpoint genes between the high-risk and low-risk groups. (b) Survival analysis of CD80. (c) Survival analysis of LDHA. (d) Survival analysis of TNFRSF4. (e) Survival analysis of TNFSF4. (f) Survival analysis of YTHDF1.

Currently, several genes are involved in the immune response, which are considered immune checkpoint genes. We investigated the expression of 38 immune checkpoint genes (listed in Supplementary Table S2) in the high and low-risk groups of the model and performed survival analysis of differentially expressed genes (DEGs). Our results showed that 31 immune checkpoint genes were DEGs (*P* < 0.05) (Figure 9(a)). Furthermore, survival analysis indicated that high expression of CD80, LDHA, TNFRSF4, and YTHDF1 was associated with poorer OS in the high-risk group compared with the low-risk group (*P* < 0.05) (Figures 9(b)–9(e)). These findings suggested that the genes in the model possibly regulate tumor immune response by modulating immune checkpoint activity, thus providing a potential target for immunotherapy.

### 4.7. Immune Infiltration Analyses

**Figure 10:**
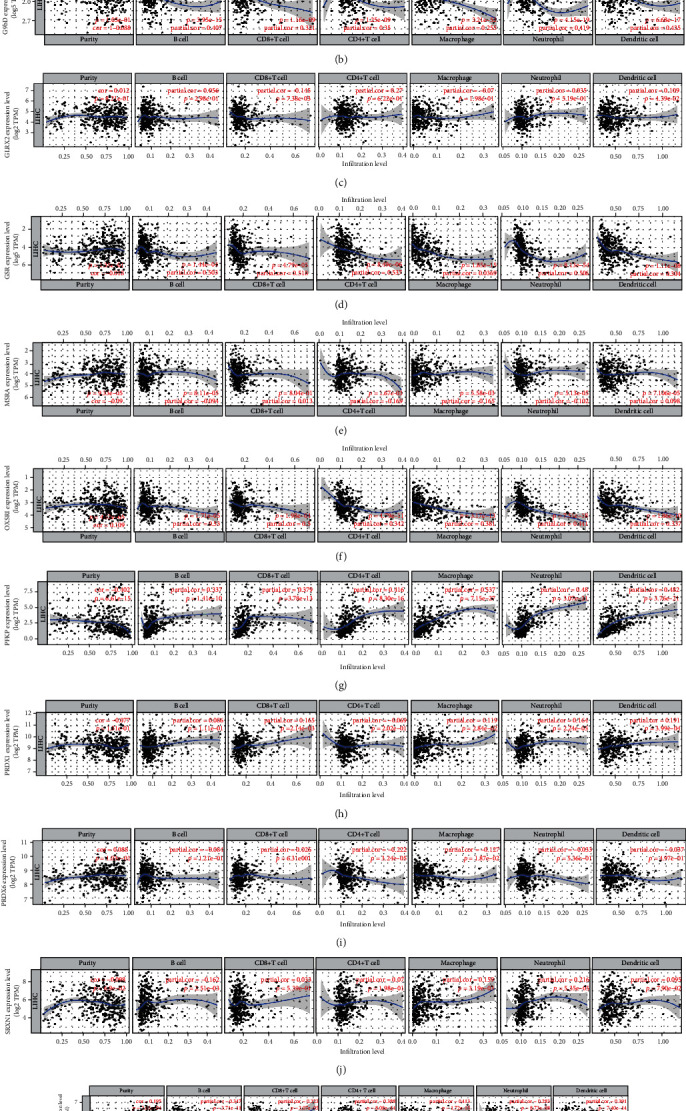
Immune infiltration analyses. (a) CDKN2D, (b) G6PD, (c) GLRX2, (d) GSR, (e) MSRA, (f) OXSR1, (g) PFKP, (h) PRDX1, (i) PRDX6, (j) SRXN1, and (k) STK25.

We further analyzed the correlation between 11 ROS-related genes and immune infiltration cells based on the TIMER online database. Our findings have shown that OXSR1, PFKP, and STK25 associate with tumor purity (all *P* < 0.05), CDKN2D correlates with B cell (cor = 0.419, *P* = 5.06*e* − 16), CD8+ T cells (cor = 0.363, *P* = 4.17*e* − 12), CD4+ T cells (cor = 0.419, *P* = 4.78*e* − 16), macrophage (cor = 0.369, *P* = 2.07*e* − 20), neutriphil (cor = 0.369, *P* = 1.46*e* − 12), and dendritic cells (cor = 0.478, *P* = 7.74*e* − 21), G6PD correlates with B cell (cor = 0.407, *P* = 3.95*e*-15), CD8+ T cells (cor = 0.321, *P* = 1.16*e* − 09), CD4+ T cells (cor = 0.32, *P* = 1.25*e* − 09), macrophage (cor = 0.522, *P* = 3.51*e* − 25), neutriphil (cor = 0.419, *P* = 4.12*e* − 16), and dendritic cells (cor = 0.432, *P* = 6.68*e* − 17), GLRX2 associates with CD8+ T cells (cor = 0.145, *P* = 7.38*e* − 03) and dendritic cells (cor = −0.109, *P* = 4.39*e* − 02), GSR associates with B cell (cor = 0.203, *P* = 1.44*e* − 04), CD8+ T cells (cor = 0.218, *P* = 4.79*e* − 05), CD4+ T cells (cor = 0.237, *P* = 8.40*e* − 06), macrophage (cor = 0.369, *P* = 1.82*e* − 12), neutriphil (cor = 0.506, *P* = 8.13*e* − 24), and dendritic cells (cor = 0.304, *P* = 1.11*e* − 08), MSRA associates with CD4+ T cells (cor = −0.169, *P* = 1.67*e* − 03) and macrophage (cor = −0.165, *P* = 2.28*e* − 03), OXSR1 correlates with B cell (cor = 0.23, *P* = 1.71*e* − 05), CD8+ T cells (cor = 0.2, *P* = 1.98*e* − 04), CD4+ T cells (cor = 0.345, *P* = 4.79*e* − 11), macrophage (cor = 0.381, *P* = 3.17*e* − 13), neutriphil (cor = 0.441, *P* = 7.21*e* − 18), and dendritic cells (cor = 0.337, *P* = 1.86*e* − 10), PFKP correlates with B cell (cor = 0.307, *P* = 1.41*e* − 10), CD8+ T cells (cor = 0.379, *P* = 3.78*e* − 13), CD4+ T cells (cor = 0.416, *P* = 8.30*e* − 16), macrophage (cor = 0.537, *P* = 1.50*e* − 27), neutriphil (cor = 0.48, *P* = 3.07*e* − 21), and dendritic cells (cor = 0.482, *P* = 3.76*e* − 21), PRDX1 correlates with B cells (cor = 0.165, *P* = 2.14*e* − 03), CD4+ T cells (cor = 0.119, *P* = 2.84*e* − 02), macrophage (cor = 0.164, *P* = 2.24*e* − 03), and neutrophil (cor = 0.191, *P* = 3.99*e* − 04), PRDX6 correlates with CD4+ T cells (cor = −0.222, *P* = 3.24*e* − 05) and macrophage (cor = −0.127, *P* = 1.87*e* − 02), SRXN1 correlates with B cells (cor = 0.162, *P* = 2.51*e* − 03), macrophage (cor = 0.159, *P* = 3.19*e* − 03), and neutrophil (cor = 0.216, *P* = 5.35*e* − 05), and STK25 correlates with B cell (cor = 0.347, *P* = 3.71*e* − 11), CD8+ T cells (cor = 0.223, *P* = 3.07*e* − 05), CD4+ T cells (cor = 0.388, *P* = 8.08*e* − 14), macrophage (cor = 0.413, *P* = 1.72*e* − 15), neutriphil (cor = 0.283, *P* = 8.73*e* − 08), and dendritic cells (cor = 0.391, *P* = 7.40*e* − 14) (Figures 10(a)–10(k)). Furthermore, we demonstrated the relationship between ROS-related genes with immune filtrating cells, and we observed that CDKN2D correlates with B cell (*r* = 0.228, *P* = 9.13*e* − 06), CD4 T cells (*r* = 0.343, *P* = 1.39*e* − 11), CD8 T cells (*r* = 0.388, *P* = 6.13*e* − 15), dendritic cells (*r* = 0.208, *P* = 5.47*e* − 05), macrophages (*r* = 0.298, *P* = 5.34*e* − 09), and neutrophils (*r* = 0.174, *P* = 0.000735), G6PD correlates with CD4 T cells (*r* = 0.406, *P* < 2.2*e* − 16), CD8 T cells (*r* = 0.188, *P* = 0.00273), dendritic cells (*r* = 0.3, *P* = 4.2*e* − 09), and macrophages (*r* = 0.126, *P* = 0.0153), GLRX2 associates with B cells (*r* = −0.192, *P* = 0.000196), CD4 T cells (*r* = 0.258, *P* = 4.99*e* − 07), and CD8 T cells (*r* = 0.116, *P* = 0.249), GSR associates with CD4 T cells (*r* = 0.196, *P* = 0.000144), CD8 T cells (*r* = −0.219, *P* = 2.06*e* − 05), dendritic cells (*r* = 0.247, *P* = 1.47*e* − 06), and neutrophils (*r* = 0.107, *P* = 0.0394), MSRA associates with CD4 T cells (*r* = −0.287, *P* = 1.93*e* − 08), CD8 T cells (*r* = 0.211, *P* = 4.17*e* − 05), macrophages (*r* = 0.114, *P* = 0.0283), and neutrophils (*r* = 0.102, *P* = 0.0489), OXSR1 correlates with B cell (*r* = −0.277, *P* = 6.18*e* − 08), CD8 T cells (*r* = −0.395, *P* = 1.35*e* − 15), dendritic cells (*r* = −0.254, *P* = 7.49*e* − 07), macrophages (*r* = −0.388, *P* = 6.84*e* − 15), and neutrophils (*r* = −0.244, *P* = 1.96*e* − 06), PFKP correlates with B cells (*r* = 0.37, *P* = 1.94*e* − 13), CD4 T cells (*r* = 0.306, *P* = 1.83*e* − 09), CD8 T cells (*r* = 0.233, *P* = 5.7*e* − 06), dendritic cells (*r* = 0.48, *P* < 2.2*e* − 16), macrophages (*r* = 0.427, *P* < 2.2*e* − 16), and neutrophils (*r* = 0.306, *P* = 1.82e-09), PRDX1 correlates with CD8 T cells (*r* = 0.277, *P* = 6.45*e* − 08), dendritic cells (*r* = 0.215, *P* = 2.85*e* − 05), and macrophages (*r* = 0.134, *P* = 0.00986), PRDX6 correlates with B cells (*r* = −0.142, *P* = 0.00609), CD4 T cells (*r* = −0.258, *P* = 4.81*e* − 07), and CD8 T cells (*r* = 0.134, *P* = 0.00952), SRXN1 correlates with dendritic cells (*r* = 0.241, *P* = 2.75*e* − 06), STK25 correlates with B cells (*r* = −0.189, *P* = 0.000253), dendritic cells (*r* = −0.223, *P* = 1.4*e* − 05), macrophages (*r* = −0.243, *P* = 2.28*e* − 06), and neutrophils (*r* = −0.291, *P* = 1.26*e* − 08) (Figure S1).

### 4.8. The Expression of 11 ROS-Related Genes in Different Immune Cells between Tumor Tissues and Matched Adjacent Tissues

**Figure 11:**
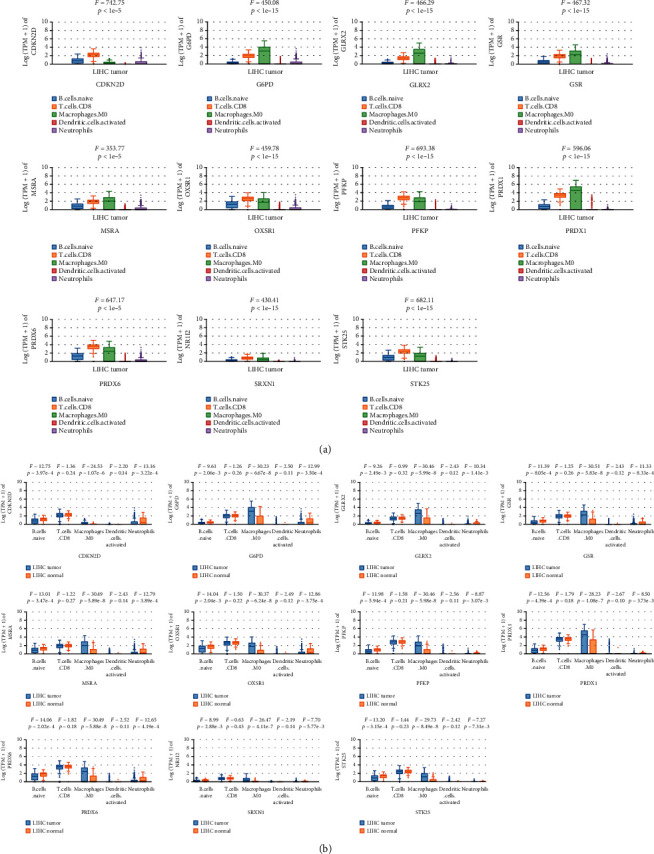
Comparison of the expression of 11 ROS-related genes in immune cells in HCC tissues and paired noncancerous tissues. (a) The expression of 11 ROS-related genes in diverse immune cells in HCC tissues. (b) The expression of 11 ROS-related genes in diverse immune cells between HCC tissues and corresponding normal tissues.

Since the expression of ROS-related genes was significantly correlated with immune-infiltrating cells, we speculated whether the levels of ROS-related gene expression were different in diverse immune cells in HCC tissues and corresponding adjacent tissues. By analyzing the GEPIA database, the findings indicated that the expression of 11 ROS-related genes was a statistical difference in HCC tissues (Figure 11(a)) (*P* < 0.05). At the same time, we also explored the expression of 11 ROS-related genes in different immune cells between HCC tissues and paired noncancerous tissues, and our results revealed that the levels of 11 ROS-related gene expressions were significantly different in B naive cells, macrophage M0, and neutrophils (Figure 11(b)) (*P* < 0.05). The above findings further demonstrated that ROS-related genes are related to tumor immunity.

### 4.9. The Correlation between the Copy Number Alterations of ROS-Related Genes and Immune-Infiltrating Cells

**Figure 12:**
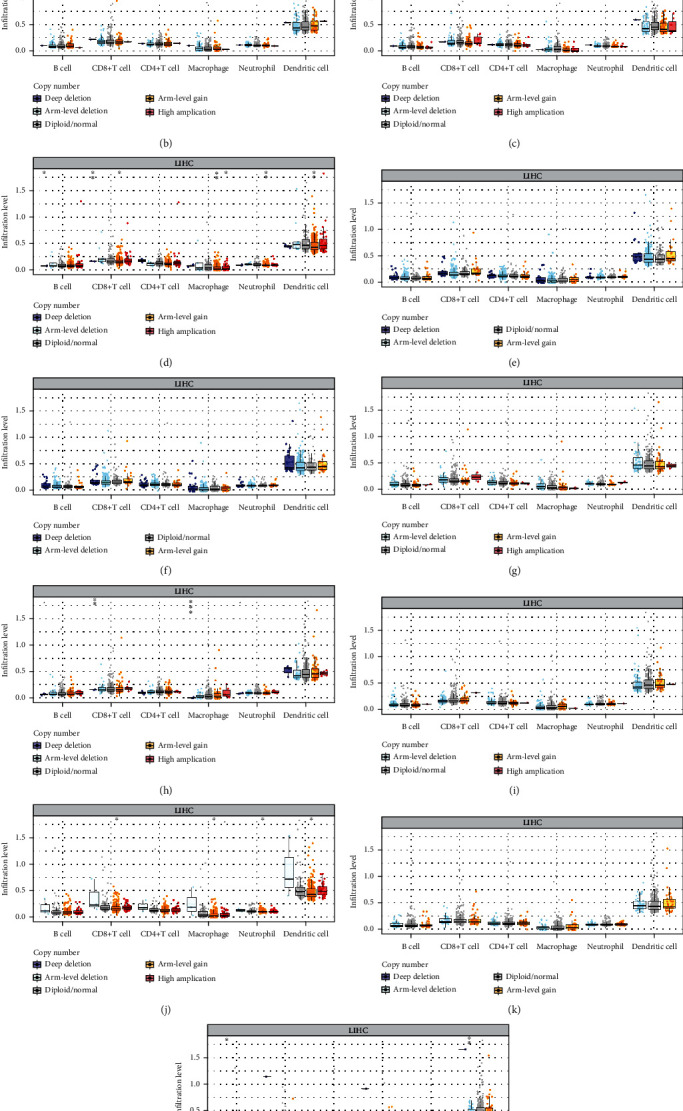
Copy number variation and immune infiltration correlated with 11 ROS-related gene expressions in HCC. (a) Copy number variation of 11 ROS-related gene expressions. (b) The relationship between copy number alterations of CDKN2D and immune infiltration. (c) The association between copy number alterations of G6PD and immune infiltration. (d) The relationship between copy number alterations of GLRK2 and immune infiltration. (e) The relationship between copy number alterations of GSR and immune infiltration. (f) The association between copy number alterations of MSRA and immune infiltration. (g) The relationship between copy number alterations of GXSR1and immune infiltration. (h) The correlation between copy number alterations of PFKP and immune infiltration. (i) The relationship between copy number alterations of PRDX1and immune infiltration. (j) The association between copy number alterations of PRDX6 and immune infiltration. (k) The relationship between copy number alterations of SRXN1 and immune infiltration. (l) The correlation between copy number alterations of STK25 and immune infiltration.

Due to somatic copy number alterations (SCNAs) which are closely correlated with different cancers, circulating tumor DNA can be used to establish genome-wide profiles of SCNAs [26, 27]. Some studies confirmed correlations between CNA signatures and cancer characteristics [28, 29]. Therefore, we investigated the copy number alterations of ROS-related genes and the relationship of CNAs with immune-infiltrating cells. Our findings manifested that the majority of genes had CNA mutations (Figure 12(a)) (*P* < 0.05), and the CNAs of G6PD, GLRX2, PFKP, PRDX1, PRDX6, and STK25 were related to immune-infiltrating cells, especially in B cells, CD8+ T cells, macrophages, neutrophils, and dendritic cells (Figures 12(b)–12(l)) (*P* < 0.05).

### 4.10. 11 ROS-Related Genes Are Correlated with Tumor Stemness, TME, and Drug Sensitivity

**Figure 13:**
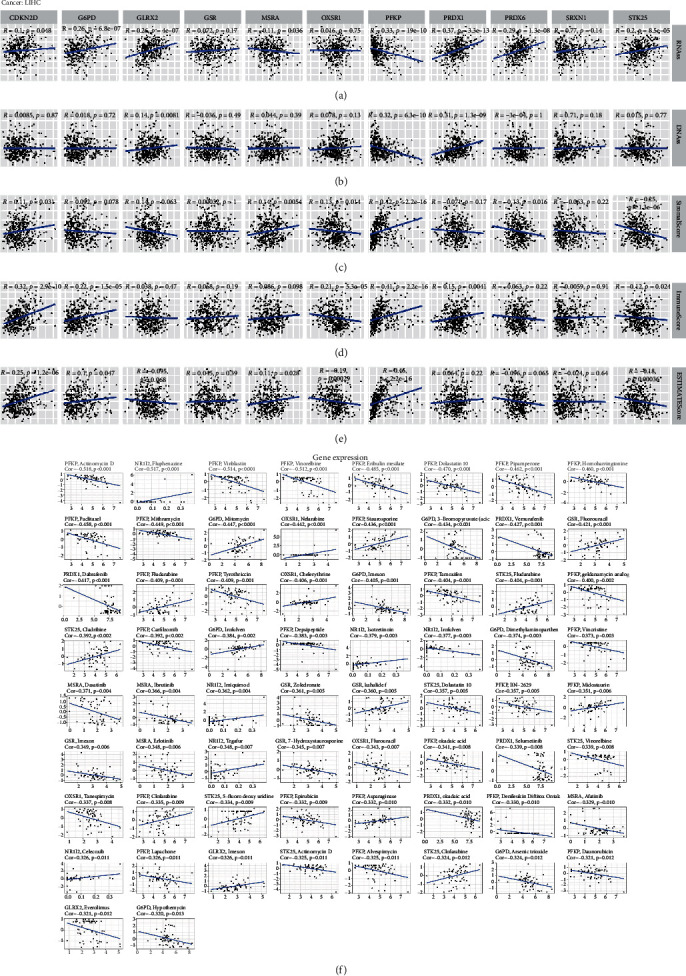
Correlation of 11 ROS-related gene expressions with tumor stemness, TME, and drug sensitivity. (a) Correlation of 11 ROS-related gene expressions with RNAss. (b) Correlation of 11 ROS-related gene expressions with DNAss. (c) Association of 11 ROS-related gene expressions with stromal score. (d) Association of 11 ROS-related gene expressions with immune score. (e) Association of 11 ROS-related gene expressions with estimate score. (f) Correlation of 11 ROS-related gene expressions with multidrug sensitivity.

Stemness represents the self-renewal, dedifferentiation with the ability to form other cell types in certain specific tissues [30]. A previous study indicated that tumor progression was not only affected by genetic changes, but also by tumor microenvironment [31]. Stemness index is associated with components of TME, and genetic variations in tumor cells can interfere with antitumor immunotherapy [32]. Our results demonstrated that the expression of CDKN2D (*R* = 0.1, *P* = 0.048), G6PD (*R* = 0.26, *P* = 6.8*e* − 07), GLRX2 (*R* = 0.26, *P* = 4*e* − 07), MSRA (*R* = −0.11, *P* = 0.036), PFKP (*R* = −0.33, *P* = 1.9*e* − 10), PRDX1 (*R* = 0.37, *P* = 3.3*e* − 13), PRDX6 (*R* = 0.29, *P* = 1.3*e* − 08), and STK25 (*R* = 0.2, *P* = 8.5*e* − 05) was related to RNAss scores, and the expression of GLRX2 (*R* = 0.14, *P* = 0.0081), PFKP (*R* = −0.32, *P* = 6.3*e* − 10), and PRDX1 (*R* = 0.31, *P* = 1.3*e* − 09) was correlated with DNAss scores (Figures 13(a) and 13(b)). Then, we further analyzed the correlation of ROS-related genes with tumor immune microenvironment, and we found that the expression of CDKN2D (*R* = 0.11, *P* = 0.031), GLRX2 (*R* = −0.14, *P* = 0.0063), MSRA (*R* = 0.14, *P* = 0.0054), OXSR1 (*R* = −0.13, *P* = 0.014), PFKP (*R* = 0.42, *P* < 2.2*e* − 16), PRDX6 (*R* = −0.13, *P* = 0.016), and STK25 (*R* = −0.25, *P* = 1.3*e* − 06) was correlated with stromal scores, the expression of CDKN2D (*R* = 0.32, *P* = 2.9*e* − 10), G6PD (*R* = 0.22, *P* = 1.5*e* − 05), OXSR1 (*R* = −0.21, *P* = 5.3*e* − 05), PFKP (*R* = 0.41, *P* < 2.2*e* − 16), PRDX1 (*R* = 0.15, *P* = 0.0041), and STK25 (*R* = −0.12, *P* = 0.024) was associated with immune scores, and the expression of CDKN2D (*R* = 0.25, *P* = 1.2*e* − 06), G6PD (*R* = 0.1, *P* = 0.047), MSRA (*R* = 0.11, *P* = 0.028), OXSR1 (*R* = −0.19, *P* = 0.00029), PFKP (*R* = 0.46, *P* < 2.2*e* − 16), and STK25 (*R* = −0.18, *P* = 0.00036) was connected with estimate scores (Figures 13(c)–13(e)). In addition, some studies indicated there was a relationship between the level of ROS and multidrug resistance in cancer [33–35]. Therefore, we investigated the association of 11 ROS-related gene expressions with chemotherapy drugs. As shown in Figure 13(f) (we took STK25 as an example), STK25 expression was correlated with clofarabine (cor = 0.392, *P* = 0.002), Actinomycin D (cor = −0.325, *P* = 0.011), 5-fluoro deoxy uridine (cor = 0.334, *P* = 0.009), Vinorelbine (cor = 0.339, *P* = 0.008), Dolastain 10 (cor = −0.357, *P* = 0.005), Cladribine (cor = −0.392, *P* = 0.002), and Fludarabine (cor = 0.404, *P* = 0.001). Collectively, the above findings indicated that ROS-related genes might be as effective targets to reduce tumor drug resistance.

### 4.11. Relationship between 11 ROS-Related Gene Expressions and Tumor Subtypes in HCC

**Figure 14:**
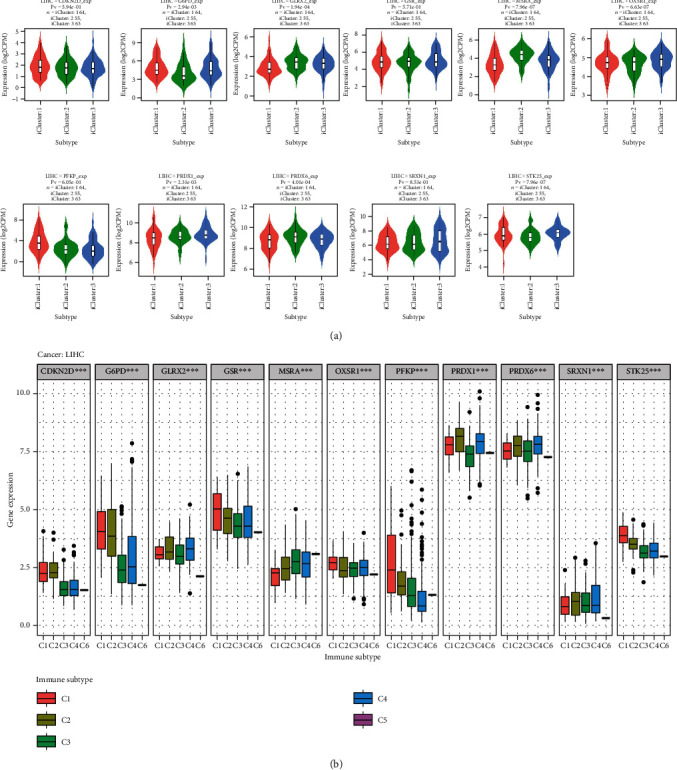
The relationship between 11 ROS-related gene expressions and subtypes of HCC. (a) The correlation of 11 ROS-related gene expressions with molecular subtypes in HCC. (b) The association of 11 ROS-related gene expressions with immune subtypes in HCC.

To further dissect the role of ROS-related genes in liver cancer, we divided liver cancer patients into three kinds of molecular subtypes based on TCGA database, namely, iCluster:1, iCluster:2, and iCluster:3, and five types of immune subtypes, namely, C1, C2, C3, C4, and C6. We explored whether the expression of ROS-related genes was a statistical difference among different subtypes. Our findings showed that the expression of G6PD, GLRX2, MSRA, PFKP, PRDX1, PRDX6, and STK25 was significantly different in three molecular subtypes (*P* < 0.05) (Figure 14(a)). We also found that the expression of CDKN2D, G6PD, GLRX2, MSRA, PFKP, PRDX1, PRDX6, and STK25 was a significant difference among five immune subtypes (*P* < 0.05) (Figure 14(b)). The above results further confirmed that ROS-related genes were involved in the occurrence and development of liver cancer.

### 4.12. 11 ROS-Related Gene Expressions and Survival Analysis in HCC

**Figure 15:**
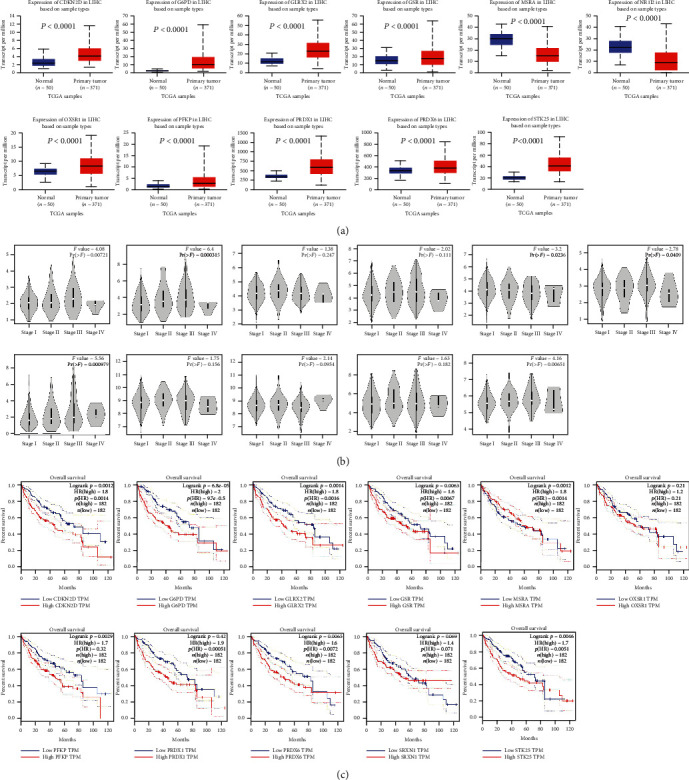
11 ROS-related gene expressions in HCC. (a) The expression of 11 ROS-related genes between HCC tissues and corresponding normal tissues. (b) Correlation of 11 ROS-related gene expressions with TNM stages in HCC. (c) Survival analysis of 11 ROS-related genes.

The aforementioned results have shown that the model can predict the survival and prognosis of liver cancer patients, and it is closely related to tumor-infiltrating cells, TME, and immune checkpoints. To further study the role of 11 ROS-related genes in liver cancer, based on the UALCAN database, we found that CDKN2D, G6PD, GLRX, GSPPFKP, PRDX1, PRDX6, SRXN, and STK25 expression was higher in liver cancer tissues than that in adjacent normal tissues, and MSRA and OXSR1 expression was lower in liver cancer tissues compared with paired noncancerous tissues (*P* < 0.05) (Figure 15(a)). Then, we also explored the correlation between the expression of 11 ROS-related genes and TNM stages in liver cancer, and the findings showed that the expression of CDKN2D, G6PD, MSRA, OXSR1, PFKP, and STK25 was connected with TNM stages (*P* < 0.05) (Figure 15(b)). Finally, survival analysis was performed based on the GEPIA and Kaplan-Meier plotter databases. The results indicated that high expression of CDKN2D (*P* = 0.0014), G6PD (*P* = 9.7*e* − 05), GLRX2 (*P* = 0.0016), GSR (*P* = 0.0067), PFKP (*P* = 0.0032), PRDX1 (*P* = 0.00051), PRDX6 (*P* = 0.0072), and STK25 (*P* = 0.0051) was associated with poor overall survival (OS) via the analysis of GEPIA cohort (Figure 15(c)). Then, we further demonstrated the relationship between the expression of 11 ROS-related genes and survival of liver cancer patients based on the Kaplan-Meier plotter database, and the findings revealed that overexpression of CDKN2D (*P* = 0.0099), G6PD (*P* = 1.1*e* − 07), GSR (*P* = 0.00019), MSRA (*P* = 0.0013), PFKP (*P* = 0.00016), PRDX1 (*P* = 0.025), SRXN1 (*P* = 0.00054), and STK25 (*P* = 0.004) was correlated with short overall survival time (Figure S2).

### 4.13. Comparison of the Protein Level of STK25 in the Tumor and Paired Noncancerous Samples

**Figure 16:**
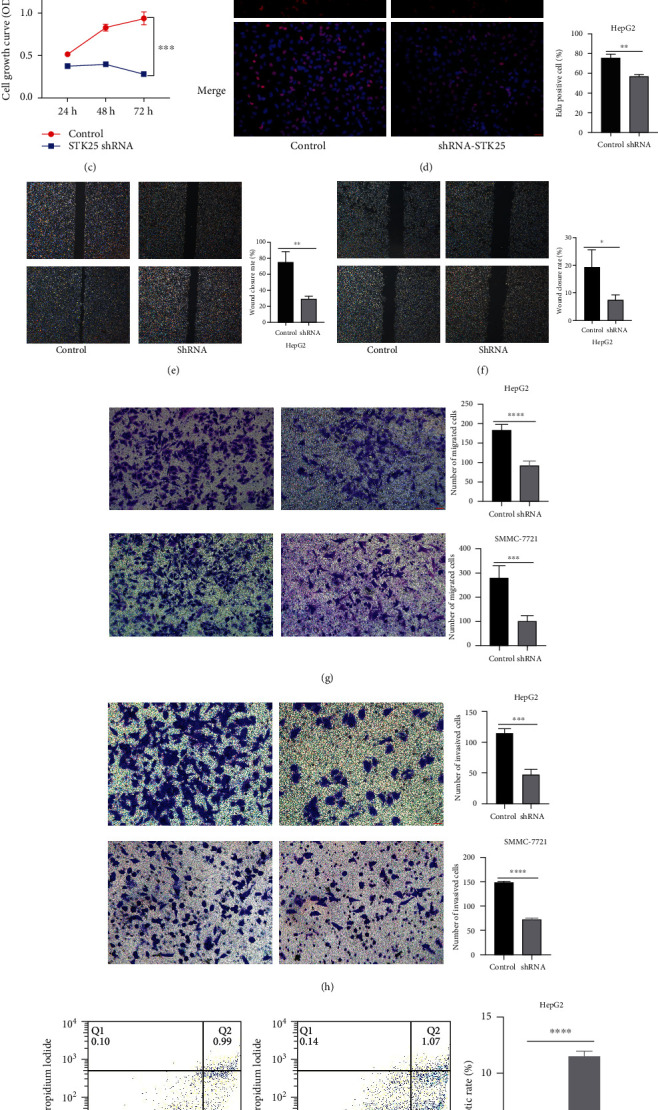
The expression and biological behavior of STK25 in liver cancer. (a) The level of STK25 expression was confirmed by GEO. (b) The protein level of STK25 expression was verified by IHC in liver cancer. Magnification, ×200. Scale bar: 50 *μ*m. (c) STK25 knockdown inhibited the proliferation capacity of HepG2 cells and SMMC-7721 via CCK8 assays. (d) STK25 knockdown suppressed the proliferation capacity of HepG2 cells via EdU assays. Magnification, ×200. Scale bar: 50 *μ*m. (e) STK25 knockdown suppressed the migration capacity of HepG2 cells via wound healing assays. Magnification, ×40. Scale bar: 200 *μ*m. (f) STK25 knockdown suppressed the migration capacity of SMMC-7721 cells via wound healing assays. Magnification, ×40. Scale bar: 200 *μ*m. (g) STK25 knockdown decreased the invasion capacity of liver cancer cells (HepG2 and SMMC-7721) via transwell invasive assays. Magnification, ×200. Scale bar: 50 *μ*m. (h) STK25 knockdown decreased the migration capacity of liver cancer cells (HepG2 and SMMC-7721) via transwell migration assays. Magnification, ×200. Scale bar: 50 *μ*m. (i) STK25 knockdown increased the apoptosis of HepG2 cells. (j) STK25 knockdown promoted the apoptosis of SMMC-7721 cells.

To further verify the expression of STK25 in liver cancer tissues and paracancerous tissues by the GEO database and clinical specimens, the expression of STK25 was confirmed based on the GEO database. The results showed that STK25 expression was higher in liver cancer tissues compared with paired noncancerous tissues (Figure 16(a)). Consistent with the results of TCGA and GEO databases, we detected STK25 expression in 9 pair tissues by performing IHC assays. Our findings revealed that the expression of STK25 was higher in the liver cancer tissues compared with the paracancerous tissues (Figure 16(b)).

### 4.14. STK25 Knockdown Promoted Apoptosis and Inhibited the Proliferation, Migration, and Invasion Capacity of Liver Cancer Cells

The analysis of public database data showed that STK25 was correlated with survival and prognosis of patients with liver cancer. We further demonstrated the role of STK25 in liver cancer by performing *in vitro* experiments. CCK8 assay revealed that STK25 knockdown significantly suppressed the proliferation of liver cancer cells (Figure 16(c)). In line with the result of CCK8 assay, EdU assays indicated that STK25 knockdown markedly decreased the proliferation capacity of liver cancer cells (Figure 16(d)). Wound healing assay and transwell migration assays were utilized to test cell migration. We observed that STK25 knockdown dramatically suppressed the migration capacity of liver cancer cells (Figures 16(e)–16(g)). In addition, transwell invasion assays were performed to estimate the invasion ability of cells, and the findings showed that STK25 knockdown significantly inhibited the invasion capacity of liver cancer cells (Figure 16(h)). A previous study demonstrated that the dysregulation of the balance between apoptosis and proliferation of cells can lead to hepatocarcinogenesis [36]. We found that STK25 knockdown significantly increased the apoptosis of liver cancer cells (Figures 16(i) and 16(j)). These results confirm that STK25 has a significant role in the progression of liver cancer.

## 5. Discussion

With advances in high-throughput sequencing technology, increasing number of prognostic biomarkers and therapeutic target are being identified. However, immune-related prognostic biomarkers of HCC are still limited. Some studies have reported that the elevated levels of ROS correlate with tumorigenesis [37, 38]. To further clarify the role of ROS-related genes in HCC, we firstly constructed a prognostic model based on ROS-related DEGs. This is the first research to explore the prognostic value of 49 ROS-related genes in HCC. Next, a prognostic model, consisting of 11 ROS-related DEGs developed through multivariate regression and LASSO Cox regression analyses. Functional enrichment analysis indicated that immune-related pathways were enriched.

ROS are by-products of cellular metabolism, including hydroxyl radicals, superoxide anions, singlet oxygen, and hydrogen peroxides [38]. Several studies have demonstrated that ROS are related to tumors [37, 39]. An increased level of ROS can impair DNA, protein, and lipids and cause genetic instability and tumorigenesis [40, 41]. Furthermore, the elevation of ROS levels can activate prosurvival signaling pathways, decrease the activation of tumor suppressor pathways, enhance glucose metabolism, and lead to tumor mutations [7, 42]. A study has reported that ROS impair mitochondrial function and oxidative stress, which led to DNA damage and hepatocarcinogenesis [43].

ROS are not only related to tumorigenesis but they also correlated with immune checkpoint inhibitors. Some studies showed that ROS-induced PD-L1 expression by regulating the JAK/STAT3 pathway [44, 45], and ROS inducers also increased the level of PD-L1 expression in tumor cells [46]. Our results also verified that most of immune checkpoint genes were differentially expressed between the high- and low-risk groups, and the overexpression of CD80, LDHA, TNFRSF4, and YTHDF1 had a shorter survival time in the high-risk group compared with the low-risk group. In addition, ROS were able to regulate immune function, cell proliferation, and epithelial-mesenchymal transition by activating profibrotic transforming growth factor-*β* (TGF-*β*), and they participated in the progression of fibrosis, tumor, and abnormal vascular function [47, 48].

To further elucidate the underlying mechanism of ROS-related genes in HCC, we conducted GO and KEGG analyses. GO analysis revealed that B cell-medicated immunity, lymphocyte-mediated immunity, immunoglobulin-mediated immune response, MHC class II receptor activity, and MHC protein complex binding were enriched. KEGG analysis indicated that the chemokine signaling pathway and cytokine-cytokine receptor interaction were enriched. Moreover, the ablation of CD20+ B cells contributed to senescence-mediated fibre regression and suppressed the TNF *α*/NF-*κ*B pathway in *Mdr2-*knockout mice [49]. In addition, this study demonstrated that the degree of B cell infiltration positively correlated with the degree of malignancy, and a high degree B cell infiltration causes a reducing in the disease-free survival rate of patients [48]. Tumor-infiltrating cytotoxic CD8+ T cells specifically suppress tumor growth and express a high level of PD1 in HCC [50]. A study also reported that PD1 exhausted CD8+ T cells in HCC [51].

Furthermore, some bioinformatics analyses were carried out to investigate 11 ROS-related genes in the model, and our results revealed that the expression of CDKN2D, G6PD, MSRA, OXSR1, and STK25 was significantly different at different stages. Survival analysis, based on the GEPIA cohort, indicated that high expression of CDKN2D, G6PD, GLRX2, GSR, PFKP, PRDX1, PRDX6, and STK25 was associated with poor OS. Additionally, our findings showed that the expression of G6PD, GLRX2, MSRA, PFKP, PRDX1, PRDX6, and STK25 was significantly different among three molecular subtypes. We also found that the expression of CDKN2D, G6PD, GLRX2, MSRA, PFKP, PRDX1, PRDX6, and STK25 was significantly different among five immune subtypes. Interestingly, the findings indicated that only G6PD and STK25 showed statistical significance. While the role of G6PD in the HCC has been investigated [52–54], the role of STK25 in liver cancer is still unclear.

STK25, as a member of the ROS family genes, is involved in lipid metabolism and tumor progression [55, 56]. In this study, the role of STK25 in liver cancer was determined through *in vitro* experiments. The findings manifested that STK25 knockdown significantly inhibited the proliferation, migration, and invasion capacity of liver cancer cells. In addition, STK25 knockdown increased the apoptosis of the cells. These results indicated that STK25, as a member of ROS family genes, played a crucial role in the progression of liver cancer.

Herein, we firstly constructed a prognostic model of ROS-related genes in HCC, and the role of STK25 in liver cancer was investigated using *in vitro* experiments. Some limitations were still in the present study. Firstly, data from public databases were not verified by our clinical samples. Secondly, all the genes involved in the study were confined to ROS-related genes, and because tumor microenvironment is highly heterogeneous, there were some limitations to the model. Finally, we did not perform *in vivo* experiments to verify the results.

In conclusion, this was the first and most comprehensive investigation of the expression of ROS-related genes and clinical characteristics in liver cancer. We firstly constructed a ROS-related prognostic model in liver cancer and confirmed the correlation of ROS-related genes with immune infiltration and immune checkpoint genes. In addition, we preliminarily investigated the role of STK25 in liver cancer. It could provide a screening instrument for HCC diagnosis and prognosis and offer a way for us to dissect the association between HCC and immunity.

## Figures and Tables

**Table 1:** Clinical features of the hepatocellular carcinoma patients in this work.

	TCGA cohort
No. of patients	365
Age (median, range)	61(16-90)
Gender (%)	
Male	246(67.4%)
Female	119(32.6%)
Stage (%)	
I	170(46.6%)
ΙΙ	84(23%)
III	83(22.7%)
IV	4(1.1%)
unknown	24(6.6%)
Survival status (%)	
Living	235(64.4%)
Death	130(35.6%)
OS days (median, range)	594(1-3675)

**Table 2:** Basic data of patients between different risk groups.

Characteristics	TCGA-LIHC cohort		*P* value
	High risk	Low risk	
Gender (%)			0.063
Female	51(28.0%)	68(37.2%)	
Male	131(72.0%)	115(62.8%)	
Age (%)			0.783
<65y	109(59.9%)	107(58.5%)	
≥65y	73(40.1%)	76(41.5%)	
TNM stage (%)			0.408
I+II	121(66.5%)	133(72.7%)	
III+IV	47(25.8%)	40(21.9%)	
unknown	14(7.7%)	10(5.5%)	
Grade (%)			<0.001
G1+G2	96(52.7%)	134(73.2%)	
G3+G4	83(45.6%)	47(25.7%)	
unknown	3(1.6%)	2(1.1%)	
Survival status (%)			<0.001
Living	101(55.5%)	134(73.2%)	
Death	81(44.5%)	49(26.8%)	

## Data Availability

All the data of this work are available from the corresponding authors upon reasonable request.

## Supplementary Materials

Supplementary 1 Figure S1: correlation of 11 ROS-related gene expressions with immune-infiltrating cells based on the TISIDB. (A) CDKN2D, (B) G6PD, (C) GLRX2, (D) GSR, (E) MSRA, (F) OXSR1, (G) PFKP, (H) PRDX1, (I) PRDX6, (J) SRXN1, and (K) STK25.

Supplementary 2 Figure S2: survival analysis of 11 ROS-related genes based on the Kaplan-Meier plotter. (A) CDKN2D, (B) G6PD, (C) GLRX2, (D) GSR, (E) MSRA, (F) OXSR1, (G) PFKP, (H) PRDX1, (I) PRDX6, (J) SRXN1, and (K) STK25.

Supplementary 3 Table S1: the list of 49 ROS-related genes.

Supplementary 4 Table S2: the list of 38 immune checkpoint genes.

Supplementary 5 Table S3: the value of the coefficient of 11 ROS-related genes.
