# Corrigendum to “System Analysis of ROS-Related Genes in the Prognosis, Immune Infiltration, and Drug Sensitivity in Hepatocellular Carcinoma”

**DOI:** 10.1155/2022/9875295

**Published:** 2022-02-21

**Authors:** Jun Hui Xu, Yong Jun Guan, Zhen Dong Qiu, Xin Zhang, Liu Liu Zi, Yu Zhou, Chen Chen, Jia Yu, Yi Chao Zhang, Wei Xing Wang

**Affiliations:** ^1^Department of Hepatobiliary Surgery, Renmin Hospital of Wuhan University, Wuhan, China; ^2^Central Laboratory, Renmin Hospital of Wuhan University, Wuhan, China; ^3^Hubei Key Laboratory of Digestive System Disease, Wuhan, China

In the article titled “*System Analysis of ROS-Related Genes in the Prognosis, Immune Infiltration, and Drug Sensitivity in Hepatocellular Carcinoma*” [1], the authors identified an error in the abstract that was introduced during the preparation of the manuscript. The last sentence should be corrected as follows:

“Furthermore, we demonstrated that STK25 knockdown could increase the proliferation, migration, and invasion capacity of HCC cells.” should be corrected to “Furthermore, we demonstrated that STK25 knockdown could decrease the proliferation, migration, and invasion capacity of HCC cells.”
