# Supplementary figures and images for: System Analysis of ROS-Related Genes in the Prognosis, Immune Infiltration, and Drug Sensitivity in Hepatocellular Carcinoma
Source: Oxid Med Cell Longev. 2021 Nov 8;2021:6485871. doi: 10.1155/2021/6485871 (PMC8593590; doi:10.1155/2021/6485871)

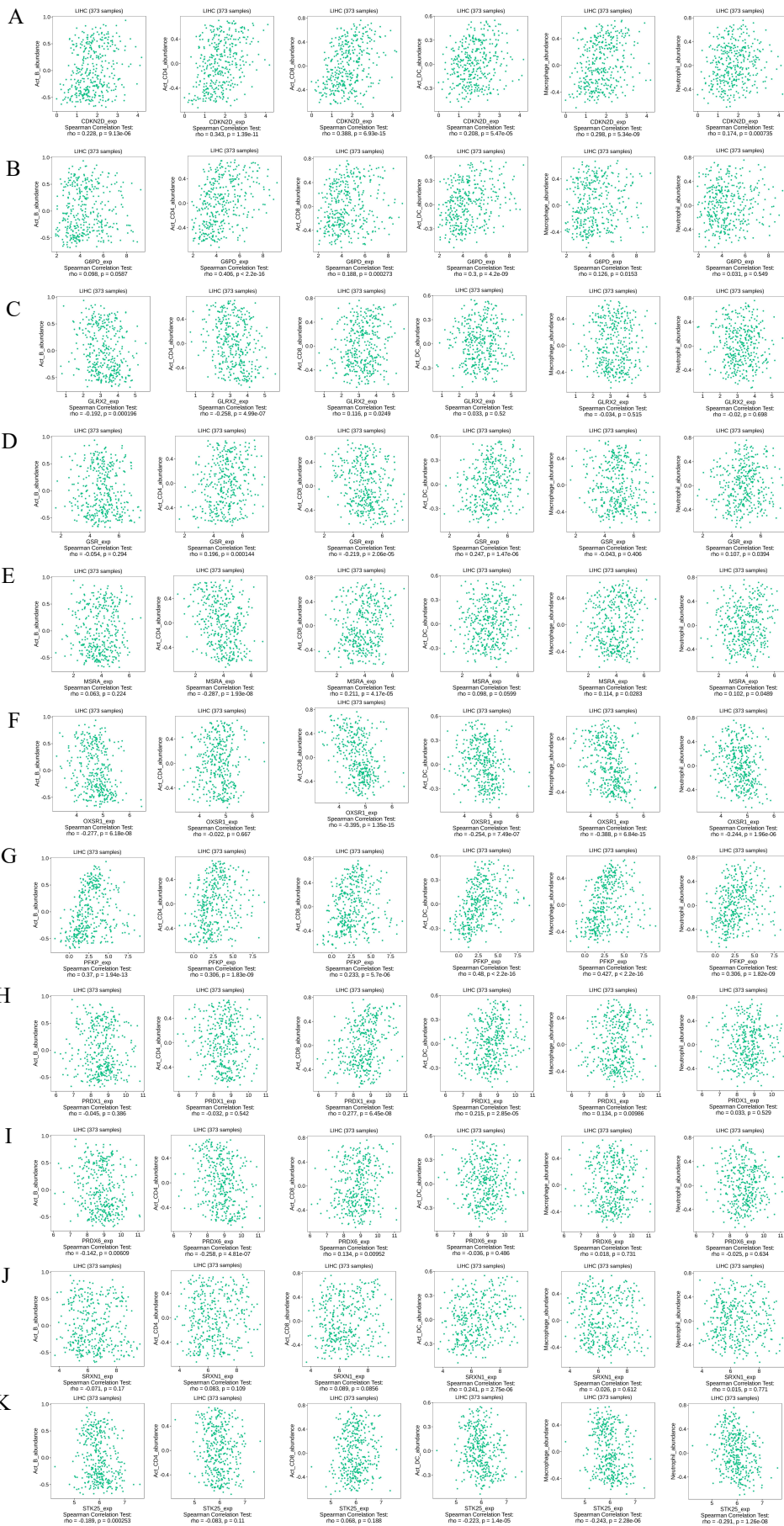

Supplement: Supplementary 1 — Figure S1: correlation of 11 ROS-related gene expressions with immune-infiltrating cells based on the TISIDB. (A) CDKN2D, (B) G6PD, (C) GLRX2, (D) GSR, (E) MSRA, (F) OXSR1, (G) PFKP, (H) PRDX1, (I) PRDX6, (J) SRXN1, and (K) STK25. [file 6485871.f1.pdf]

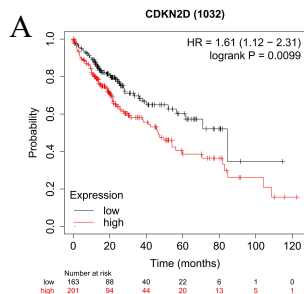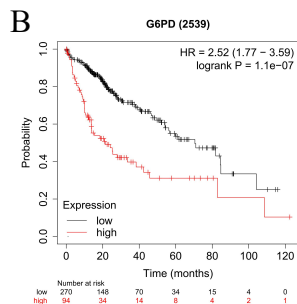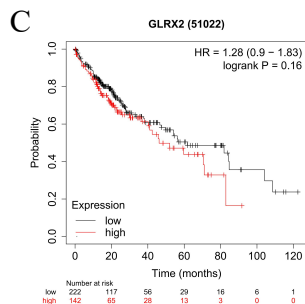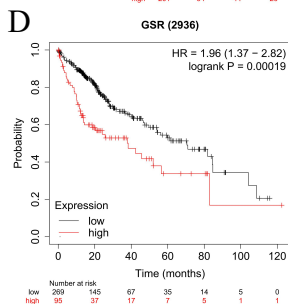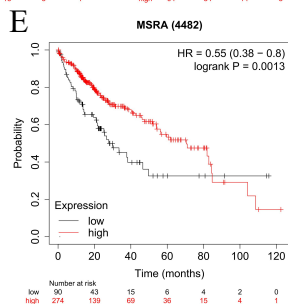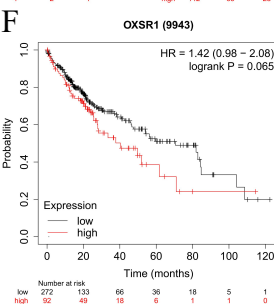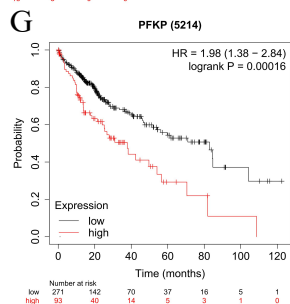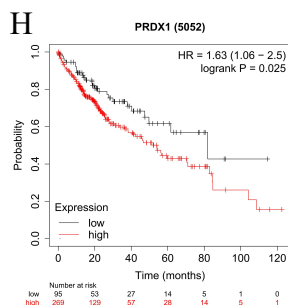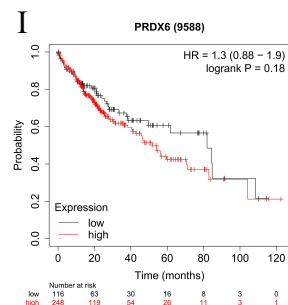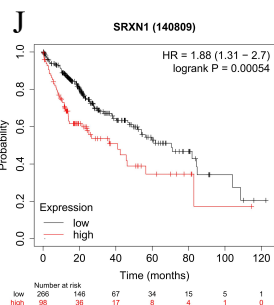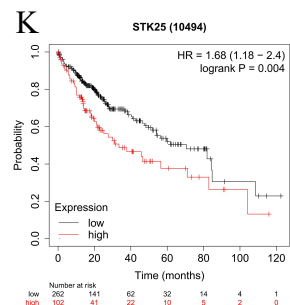

Supplement: Supplementary 2 — Figure S2: survival analysis of 11 ROS-related genes based on the Kaplan-Meier plotter. (A) CDKN2D, (B) G6PD, (C) GLRX2, (D) GSR, (E) MSRA, (F) OXSR1, (G) PFKP, (H) PRDX1, (I) PRDX6, (J) SRXN1, and (K) STK25. [file 6485871.f2.pdf]
